# A new prognostic instrument to predict the probability of developing new cerebral metastases after radiosurgery alone

**DOI:** 10.1186/1748-717X-9-215

**Published:** 2014-09-20

**Authors:** Stefan Huttenlocher, Liesa Dziggel, Dagmar Hornung, Oliver Blanck, Steven E Schild, Dirk Rades

**Affiliations:** Department of Radiation Oncology, University of Lübeck, Ratzeburger Allee 160, Lübeck, 23538 Germany; Department of Radiation Oncology, University Medical Center Eppendorf, Hamburg, Germany; CyberKnife Centre Northern Germany, Güstrow, Germany; Department of Radiation Oncology, Mayo Clinic, Scottsdale, AZ USA

**Keywords:** Cerebral metastases, Radiosurgery, Freedom from new cerebral metastases, Prognostic instrument, Whole-brain irradiation

## Abstract

**Background:**

Addition of whole-brain irradiation (WBI) to radiosurgery for treatment of few cerebral metastases is controversial. This study aimed to create an instrument that estimates the probability of developing new cerebral metastases after radiosurgery to facilitate the decision regarding additional WBI.

**Methods:**

Nine characteristics were investigated for associations with the development of new cerebral metastases including radiosurgery dose (dose equivalent to <20 Gy *vs*. 20 Gy *vs*. >20 Gy for tumor cell kill, prescribed to the 73-90% isodose level), age (≤60 *vs*. ≥61 years), gender, Eastern Cooperative Oncology Group performance score (0-1 *vs*. 2), primary tumor type (breast cancer *vs.* non-small lung cancer *vs.* malignant melanoma *vs.* others), number/size of cerebral metastases (1 lesion <15 mm *vs*. 1 lesion ≥15 mm *vs.* 2 or 3 lesions), location of the cerebral metastases (supratentorial alone *v*s. infratentorial ± supratentorial), extra-cerebra metastases (no *vs*. yes) and time between first diagnosis of the primary tumor and radiosurgery (≤15 *v*s. >15 months).

**Results:**

Number of cerebral metastases (p = 0.002), primary tumor type (p = 0.10) and extra-cerebral metastases (p = 0.06) showed significant associations with development of new cerebral metastases or a trend, and were integrated into the predictive instrument. Scoring points were calculated from 6-months freedom from new cerebral metastases rates. Three groups were formed, group I (16-17 points, N = 47), group II (18-20 points, N = 120) and group III (21-22 points, N = 47). Six-month rates of freedom from new cerebral metastases were 36%, 65% and 80%, respectively (p < 0.001). Corresponding rates at 12 months were 27%, 44% and 71%, respectively.

**Conclusion:**

This new instrument enables the physician to estimate the probability of developing new cerebral metastases after radiosurgery alone. Patients of groups I and II appear good candidates for additional WBI in addition to radiosurgery, whereas patients of group III may not require WBI in addition to radiosurgery.

## Background

For patients with a limited number of cerebral metastases, radiosurgery has been suggested to result in better local control rates than resection of the lesions [1]. This has also been shown for patients presenting with solitary metastasis [2, 3]. Furthermore, radiosurgery is non-invasive lacking surgical or anesthesia related risks. Therefore, many patients with a very few cerebral metastases receive radiosurgery. It is not yet clear whether radiosurgery should be supplemented by the addition of whole-brain irradiation (WBI). On one hand, the combined approach was shown to result in worse neurocognitive functions after four months than radiosurgery alone in a small randomized trial (4). On the other hand, radiosurgery plus WBI leads to improved intra-cerebral control at one year in comparison to radiosurgery alone [4, 5]. Several authors have attributed intra-cerebral recurrence as the most important factor associated with post-treatment neurocognitive decline [5–7]. In order to make an appropriate decision for or against the addition of WBI, a prognostic instrument would be helpful that predicts a patient’s probability of developing new cerebral metastases outside those lesions treated with radiosurgery. This study intended to develop just such an instrument based on a careful analysis of a cohort of 214 patients who received radiosurgery alone for very few cerebral metastases.

## Patients and methods

### Patients and treatment

A total of 214 patients who had received radiosurgery alone for treatment of 1-3 cerebral metastases from solid tumors included in this retrospective study. In the entire cohort, 186 patients were irradiated with linear accelerator based radiosurgery (Siemens, Medical Systems, Concord, CA, USA and Varian Medical Systems, Palo Alto, CA, USA); 28 patients had received Cyberknife radiosurgery (Accuray, Sunnyvale, CA, USA). The study was indicated at the responsible local ethics committee (University of Lübeck). It was performed in compliance with the Helsinki Declaration. The patients generally had imaging follow-up data available at about six weeks, about three months, about six months and about twelve months following radiosurgery, as well as in case of progressive or new symptoms. The following nine characteristics were analyzed for freedom from new brain metastases distant from the lesions treated with radiosurgery: radiosurgery dose (dose equivalent to <20 Gy *vs*. 20 Gy *vs*. >20 Gy for tumor cell kill (α/β-ratio = 10 Gy), prescribed to the 73-90% isodose level), age (≤60 *vs*. ≥61 years), gender, Eastern Cooperative Oncology Group performance score (ECOG-PS 0-1 *vs*. ECOG-PS 2), primary tumor type (breast cancer *vs.* non-small lung cancer *vs.* malignant melanoma *vs.* other tumors), number/size of cerebral metastases (1 lesion <15 mm *vs*. 1 lesion ≥15 mm *vs.* 2 or 3 lesions), location of the cerebral metastases (supratentorial alone *v*s. infratentorial ± supratentorial), extra-cerebra metastases (no *vs*. yes) and time between first diagnosis of the primary tumor and administration of radiosurgery (≤15 *v*s. >15 months). Other tumors included renal cell carcinoma (N = 16), colorectal cancer (N = 13), small cell lung cancer (N = 6), upper gastrointestinal cancers (N = 3), gynecological cancers (N = 2), and cancer of unknown primary (N = 2).

### Statistics

The univariate analysis of freedom from new cerebral metastases was done with the Kaplan-Meier method and the Wilcoxon test. Characteristics that achieved significance (*p* < 0.05) or showed a trend (*p* ≤ 0.10) were included in the prognostic instrument designed to predict the probability of developing new cerebral metastases distant from the lesions treated with radiosurgery.

## Results

In the univariate analysis of freedom from new cerebral metastases, the number of cerebral metastases achieved significance (*p* = 0.002). Primary tumor type (*p* = 0.10) and extra-cerebral metastases (*p* = 0.06) showed a trend (Table 1). These three characteristics were included in the prognostic instrument. Scoring points were calculated by dividing the freedom from new cerebral metastases rate at six months following radiosurgery (given in %) by 10 (Table 2). The prognostic score for each patient was derived from adding the scores of each of the three characteristics. Since the score for one lesion was 7 points regardless of the lesion size, 1 lesion <15 mm and 1 lesion ≥15 mm were combined (Table 2). The prognostic scores ranged from 16 to 22 points.

**Table 1:** **Freedom from new brain metastases at 6 months following radiosurgery**

	Rates of freedom from new brain metastases (%)	***p***
**Radiosurgery dose**		
<20 Gy (N = 69)	60	
20 Gy (N = 124)	61	
>20 Gy (N = 21)	67	0.70
**Age**		
≤60 years (N = 99)	68	
≥61 years (N = 115)	56	0.49
**Gender**		
Female (N = 112)	63	
Male (N = 102)	60	0.51
**ECOG performance score**		
0-1 (N = 158)	62	
2 (N = 56)	62	0.75
**Primary tumor type**		
Breast cancer (N = 21)	80	
Non-small cell lung cancer (N = 72)	67	
Malignant melanoma (N = 79)	54	
Other tumors(N = 42)	57	0.10
**Number of cerebral metastases**		
1 lesion <15 mm (N = 132)	70	
1 lesion ≥15 mm (N = 55)	71	
2 or 3 lesions (N = 82)	48	0.002
**Location of cerebral metastases**		
Supratentorial alone (N = 179)	63	
Infratentorial ± supratentorial (N = 35)	55	0.49
**Extra-cerebral metastases**		
No (N = 88)	69	
Yes (N = 126)	56	0.06
**Time tumor diagnosis to radiosurgery**		
≤15 months (N = 95)	63	
>15 months (N = 119)	61	0.90

ECOG = Eastern Cooperative Oncology Group.

**Table 2:** **Characteristics included in the prognostic instrument: Freedom from new brain metastases at 6 months following radiosurgery and the corresponding scoring points**

	Rates of freedom from new brain metastases (%)	Scoring points
**Primary tumor type**		
Breast cancer (N = 21)	80	8
Non-small cell lung cancer (N = 72)	67	7
Malignant melanoma (N = 79)	54	5
Other tumors(N = 42)	57	6
**Number of cerebral metastases**		
1 lesion (N = 132)	70	7
2 or 3 lesions (N = 82)	48	5
**Extra-cerebral metastases**		
No (N = 88)	69	7
Yes (N = 126)	56	6

The 6-month rates of freedom from new cerebral metastases were 34% for 16 points, 38% for 17 points, 59% for 18 points, 67% for 19 points, 69% for 20 points, 80% for 21 points and 83% for 22 points, respectively (*p* = 0.002). According to these prognostic scores, three different groups were designed: group I (16-17 points, N = 47), group II (18-20 points, N = 120) and group III (21-22 points, N = 47). The 6-month rates of freedom from new cerebral metastases of the three groups were 36%, 65% and 80%, respectively (*p* < 0.001). The corresponding rates at 12 months were 27%, 44% and 71%, respectively. Of the patients developing new cerebral metastases, 26% in group I, 45% in group II and 62% in group III, respectively, were possible candidates for a second course of radiosurgery.

## Discussion

Many patients presenting with very few cerebral metastases receive radiosurgery, since radiosurgery appears to result in better local control of the treated lesions than neurosurgical resection [8, 9]. In a matched-pair analysis that compared resection followed by WBI to WBI plus a radiosurgery boost in patients with one to three cerebral metastases, outcomes were better in the WBI plus radiosurgery group. One-year survival rates were 56% after WBI plus radiosurgery and 47% after resection plus WBI, respectively (*p* = 0.034). One-year intra-cerebral control rates were 66% and 50%, respectively (*p* = 0.003), and one-year local control rates of the treated lesions were 82% and 66%, respectively (*p* = 0.006) [1]. In another retrospective study of 152 patients, similar results were observed with respect to local control of a single lesion [3]. However, the results of resection followed by WBI may be improved with the addition of a radiation boost to the metastatic sites [10].

Controversy exists as to whether the addition of WBI to radiosurgery provides a benefit for patients. WBI may be associated with an increase in treatment-related neurocognitive deficits. This was demonstrated in a randomized trial [4]. That study was stopped after an interim analysis of 58 patients because the decline in neurocognitive function at four months was significantly greater in the radiosurgery plus WBI group compared to the radiosurgery alone group. However, at 12 months, intra-cerebral control was significantly better in the patients who received the combined approach (73% *vs.* 27%, *p* < 0.001). Unfortunately, no evaluation of neurocognitive function was performed at 12 months. Neurocognitive function would be expected to have been worse in the radiosurgery alone group at 12 months, because intra-cerebral recurrence and not additional WBI is reported as the most important cause of neurocognitive dysfunction. In the prospective study of Aoyama *et al*., neurocognitive function was preserved in 79% of patients after radiosurgery plus WBRT and in 53% of patients after radiosurgery alone, respectively, after one year, and in 79% and 43% of patients, respectively after two years [5]. Improved intra-cerebral control with the addition of WBI has also been reported in a retrospective study of 144 patients with 1-3 cerebral metastases [11]. One-year intra-cerebral control rates were 66% and 51%, respectively (*p* = 0.015) [11]. However, these results were not translated into significantly better survival. This has been an argument against the addition of WBI. New cerebral metastases after radiosurgery alone may be treated with another course of radiotherapy or with neurosurgical resection of the metastases.

Thus, the question whether WBI should be added to radiosurgery has not yet been answered properly. A prognostic instrument that allows the physician to estimate the probability of developing new cerebral metastases outside the areas treated with radiosurgery may help when making the decision whether to add WBI.

This study provides just such an instrument. Based on three characteristics (primary tumor type, number of cerebral metastases, extra-cerebral metastases) three groups were formed. These three prognostic factors were also significantly associated with distant brain failure in a recently reported retrospective series of 464 patients treated with Gamma Knife radiosurgery alone [12]. In another recently published retrospective study of 361 patients receiving Linac-based radiosurgery alone, age, number of cerebral lesions, performance status and lesion size were significantly associated with the risk of regional failure [13].

In the majority of the patients belonging to group I, new distant cerebral metastases developed within six months following radiosurgery alone. Only 26% of patients in this group were possible candidates for a second course of radiosurgery. Therefore, patients of this group likely would benefit from the addition of WBI to radiosurgery. In group II, about one third of the patients developed new cerebral metastases within six months, and more than half of the group within 12 months. Less than 50% of these patients were possible candidates for repeat radiosurgery. Thus, also in this group, the addition of WBI should be considered. In group III, only 20% of the patients developed new cerebral metastases within six months following radiosurgery. Also the rate of new lesions at 12 months was relatively low at about 30%. Sixty-two percent of these patients appeared suitable for a second course of radiosurgery. Therefore, patients belonging to this group may not require WBI in addition to radiosurgery. Because this study included different primary tumor types, the predictive instrument might be generalized to these entities. However, since the patient cohort consisted mainly of patients with melanoma breast cancer or non-small cell lung cancer, the predictive score mainly applies to these entities. When considering the findings of this study, its retrospective nature should be taken into account. Retrospective studies always bear the risk of hidden selection biases and suffer from non-standardized follow-up. Furthermore, it is unclear whether all patients developing new cerebral lesions do require salvage treatment. In the study of Zindler *et al.*, only 25% of patients needed salvage treatment following radiosurgery alone, although 41% of patients had developed new cerebral lesions within 12 months [14].

In conclusion, this new prognostic instrument enables the physician to estimate the probability that a patient will develop new cerebral metastases after radiosurgery alone. Patients belonging to groups I and II appear good candidates for WBI in addition to radiosurgery. Patients of group III may not require WBI in addition to radiosurgery. Because not all factors included in the predictive instrument did achieve significance, subsequent validation in an independent patient cohort is required. A simplification of the current instrument by using a model, which is based on numbers of unfavorable characteristics instead of weighed scores, may also be considered, when the current instrument will be validated. However, such a simpler model may not properly reflect the weight of each prognostic factor.
